# Cutaneous Metastasis Presenting as Vesicular-Like Lesions

**DOI:** 10.5826/dpc.1103a61

**Published:** 2021-07-08

**Authors:** Ravikumar Mudugal, Anupama Bains, Anil Budania, Meenakshi Rao

**Affiliations:** 1 Department of Dermatology, All India Institute of Medical Sciences, Jodhpur, Rajasthan, India; 2 Department of Pathology, All India Institute of Medical Sciences, Jodhpur, Rajasthan, India

**Keywords:** Cutaneous metastasis, vesicular-like

## Case Presentation

**Figure 1:**
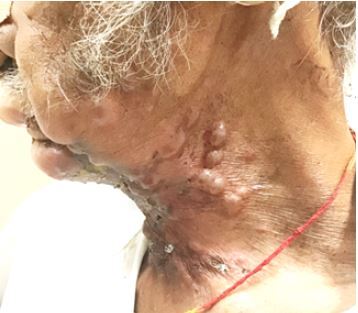
Multiples vesicular-like lesions over the anterior and lateral side of the neck.

**Figure 2:**
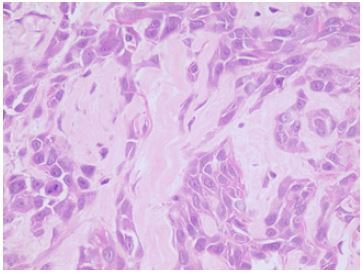
Histopathological examination (H&E, ×40) showed tumor in dermis with squamoid morphology, polygonal cells, abundant eosinophilic cytoplasm, and vesicular nucleus.

A 76-year-old man presented with vesicular-like lesions covering the submandibular area and the sides of the neck (Figure 1). The patient was a known case of squamous cell carcinoma affecting the floor of the mouth. Histopathology of the lesions showed a tumor in the dermis with squamoid morphology, polygonal cells, abundant eosinophilic cytoplasm, and vesicular nucleus (Figure 2). On immunohistochemistry, cells were positive for p63 and p40 markers. Final diagnosis of cutaneous metastasis, secondary to squamous cell carcinoma, was made.

## Teaching Point

Cutaneous metastases generally present as solitary or multiple hard nodules. Other atypical presentations such as morphea-like, erysipelas-like, alopecia neoplastica, zosteriform metastases, etc., are rarely seen [1]. Vesicobullous metastasis is rare and has been previously described in breast cancers and melanomas. In our case, because of the vesicular-like lesions, the disease mimicked disseminated herpes zoster, radiotherapy induced bullous pemphigoid, hence awareness regarding such presentation is important.
